# Starving cancer from the outside and inside: separate and combined effects of calorie restriction and autophagy inhibition on Ras-driven tumors

**DOI:** 10.1186/s40170-016-0158-4

**Published:** 2016-09-16

**Authors:** Laura M. Lashinger, Ciara H. O’Flanagan, Sarah M. Dunlap, Audrey J. Rasmussen, Shannon Sweeney, Jessie Yangxiang Guo, Alessia Lodi, Stefano Tiziani, Eileen White, Stephen D. Hursting

**Affiliations:** 1 Department of Nutritional Sciences, University of Texas at Austin, Austin, TX 78723 USA; 2 Department of Nutrition, University of North Carolina, Chapel Hill, NC 27517 USA; 3 Rutgers Cancer Institute of New Jersey, 195 Little Albany Street, New Brunswick, NJ 08903 USA; 4 Department of Molecular Biology and Biochemistry, Rutgers University, Piscataway, NJ 08854 USA; 5 Department of Chemical Biology, Rutgers Ernest Mario School of Pharmacy, Piscataway, NJ 08854 USA; 6 Department of Nutrition, University of North Carolina at Chapel Hill, 2115 Michael Hooker Research Center, Campus Box 7461, Chapel Hill, NC 27599 USA

**Keywords:** Calorie restriction, Autophagy, Metabolomics, Nutrient stress, Cancer

## Abstract

**Background:**

Calorie restriction (CR) prevents obesity and exerts anticancer effects in many preclinical models. CR is also increasingly being used in cancer patients as a sensitizing strategy prior to chemotherapy regimens. While the beneficial effects of CR are widely accepted, the mechanisms through which CR affects tumor growth are incompletely understood. In many cell types, CR and other nutrient stressors can induce autophagy, which provides energy and metabolic substrates critical for cancer cell survival. We hypothesized that limiting extracellular and intracellular substrate availability by combining CR with autophagy inhibition would reduce tumor growth more effectively than either treatment alone.

**Results:**

A 30 % CR diet, relative to control diet, in nude mice resulted in significant decreases in body fat, blood glucose, and serum insulin, insulin-like growth factor-1, and leptin levels concurrent with increased adiponectin levels. In a xenograft model in nude mice involving H-Ras^G12V^-transformed immortal baby mouse kidney epithelial cells with (*Atg5*^*+/+*^) and without (*Atg5*^−*/*−^) autophagic capacity, the CR diet (relative to control diet) genetically induced autophagy inhibition and their combination, each reduced tumor development and growth. Final tumor volume was greatest for *Atg5*^*+/+*^ tumors in control-fed mice, intermediate for *Atg5*^*+/+*^ tumors in CR-fed mice and *Atg5*^−*/*−^ tumors in control-fed mice, and lowest for *Atg5*^−*/*−^ tumors in CR mice. In *Atg5*^*+/+*^ tumors, autophagic flux was increased in CR-fed relative to control-fed mice, suggesting that the prosurvival effects of autophagy induction may mitigate the tumor suppressive effects of CR. Metabolomic analyses of CR-fed, relative to control-fed, nude mice showed significant decreases in circulating glucose and amino acids and significant increases in ketones, indicating CR induced negative energy balance. Combining glucose deprivation with autophagy deficiency in *Atg5*^−*/*−^ cells resulted in significantly reduced in vitro colony formation relative to glucose deprivation or autophagy deficiency alone.

**Conclusions:**

Combined restriction of extracellular (via CR in vivo or glucose deprivation in vitro) and intracellular (via autophagy inhibition) sources of energy and nutrients suppresses Ras-driven tumor growth more effectively than either CR or autophagy deficiency alone. Interventions targeting both systemic energy balance and tumor-cell intrinsic autophagy may represent a novel and effective anticancer strategy.

## Background

Calorie restriction (CR), a nutritionally replete dietary regimen that reduces calorie intake without incurring malnutrition, extends lifespan in species from yeast to mammals, and in rodents delays the onset of multiple age-associated diseases, including cancer [1–8]. CR improves health and survival in rhesus monkeys, including decreased risks of diabetes, neurodegeneration, and cancer [9–12]. Though it is not yet known whether CR can extend lifespan in humans, CR improves markers of cardiovascular aging and skeletal decline, and decreases inflammatory and endocrine markers typically associated with age-related diseases such as cancer [13–18], suggesting that the beneficial effects of CR on metabolism and chronic disease risk in rodent and primate models may also extend to humans. In addition, CR can reprogram several cancer-related signaling pathways and increase the susceptibility of some cancer cells to chemotherapeutic agents [19, 20].

CR redirects metabolic investment from cellular replication toward maintenance and survival [19, 20]. Many of the beneficial effects of CR may be attributed to signaling through the AMP-activated protein kinase/mammalian target of rapamycin (AMPK/mTOR) axis [19, 20]. This system integrates intracellular and extracellular signals and enables cells to adapt to environmental changes, such as nutrient or growth factor availability. Activation of mTOR promotes cell survival, proliferation, and protein translation pathways. In low nutrient conditions, AMPK acts as a molecular sensor activating fatty acid oxidation and glucose uptake, inhibiting mTOR signaling, and regulating synthesis of proteins, lipids, glycogen, and nucleotides. Many cancers can hijack mTOR/AMPK signaling to promote their growth in changing external circumstances [21]. CR suppresses mTOR and increases AMPK activity in many tissues and organs [22]. A key target of the AMPK/mTOR pathway is macroautophagy (hereafter referred to as autophagy), an evolutionarily conserved, tightly controlled pathway in which proteins, macronutrients, and organelles are sequestered in double-membraned vesicles and lysosomally degraded. Autophagy is a homeostatic process, providing a conduit for clearance of damaged organelles and aggregated proteins. Autophagy can also function to promote cell survival during times of nutrient deprivation, as digested molecules can be recycled to provide energy and metabolic precursors until external nutrients are replenished [23]. Autophagy is inhibited by mTOR activation, while AMPK induces autophagy during low nutrient availability [24].

The role of autophagy in cancer is complex and likely context-dependent. Autophagy can be tumor suppressive, in particular during tumor initiation [25], and is regulated by several oncogenes including Myc, Akt, PI3-kinase, and Ras [26]. Oncogenic mutations in Ras are known to induce autophagy, and many Ras-driven tumors are dependent on autophagy [27–29], likely to provide an ancillary source of energy to rapidly proliferating cancer cells. In established tumors, autophagy induction can be prosurvival, allowing cancer cells to overcome nutrient deprivation, hypoxic conditions, oxidative and metabolic stress, and even chemotherapy [27, 30, 31].

The anti-aging effects of CR in *Caenorhabditis elegans* are dependent on autophagy [32], though the interaction between CR and autophagy in mammals is less clear [33], and manipulation of autophagy in combination with CR on tumor development has not been previously investigated. We hypothesized that limiting extracellular and intracellular substrate availability by combining CR with autophagy inhibition would reduce in vivo tumor growth more effectively than either treatment alone. Using an H-Ras-transformed xenograft tumor model, we show here that CR (in association with a metabolic shift away from glucose utilization and toward fatty acid oxidation) and autophagy deficiency each suppresses tumor growth. Moreover, combining CR with autophagy deficiency further reduces tumor growth relative to either intervention alone.

## Methods

### Cell lines

Immortalized baby mouse kidney epithelial cells (iBMK) were derived from *Atg5*^*+/+*^ or *Atg5*^−*/*−^ mice and maintained as described previously [28, 33, 34]. Briefly, iBMK cells were immortalized by E1A and dominant-negative p53 expression. iBMK cells were then stably transfected with a H-Ras^G12V^ expression construct, and a p-tFl-RFP-tandem tagged microtubule-associated protein1 light chain 3 (LC3) expression plasmid [28]. Cells were cultured in DMEM (GIBCO/Invitrogen) supplemented with 10 % fetal bovine serum (FBS), and 1 % penicillin/streptomycin (Sigma-Aldrich) at 38.5 °C with 8.5 % CO_2_. For colony formation assay, 500 cells were seeded in triplicate into each well of a six-well plate and incubated for 10 days in DMEM containing 1, 5, or 10 mM glucose. Colonies were fixed for 5 min with 100 % methanol and stained with 0.5 % crystal violet in 50 % methanol.

### Mice, diets, and experimental design

All mouse experimentation was conducted in the Animal Resource Center at the University of Texas at Austin in accordance with the recommendations outlined in the Guide for the Care and Use of Laboratory Animals set forth by the National Institutes of Health. All experimentation was approved by the Institutional Animal Care and Usage Committee at the University of Texas at Austin. All efforts were taken to minimize animal suffering.

Female athymic nude mice (4–6 weeks of age; *n* = 94) were singly housed and randomly selected to receive one of the following diets (*n* = 47/diet group): (1) control diet consumed ad libitum (#D12450B, a modified AIN-76A semipurified diet with 10 kcal% fat) that generates an overweight phenotype or (2) 30 % calorie-restricted diet (CR, #D0302702) consumed in daily aliquots that generate a lean phenotype. The CR diet is based on the control diet but modified such that when fed as a daily aliquot provides 70 % of the total caloric intake but 100 % of all vitamins, minerals, fatty acids, and amino acids. Both diets were from Research Diets (New Brunswick, NJ).

Following 16 weeks on either control or CR diet, a subset of mice (*n* = 19/diet group) were fasted for 6 h, anesthetized with CO_2_, and whole blood was collected by cardiac puncture for fasting glucose assessment and to obtain serum samples for circulating hormone and growth factor analysis. Mice were subsequently killed by cervical dislocation. Blood was allowed to coagulate (30 min at room temperature), centrifuged at 9300×*g* for 5 min, and serum was collected and stored at −80 °C. Carcasses were analyzed for body composition parameters using dual energy X-ray absorptiometry (GE Lunar PIXImus; Fitchburg, WI).

The remaining 28 mice for each diet group were randomly selected for subcutaneous injection (left flank) with 5 × 10^4^ autophagy-competent *Atg5*^*+/+*^*;Hras*^*V12*^ iBMK (*Atg5*^*+/+*^) cells (*n* = 14 mice) or autophagy-deficient *Atg5*^−*/*−^*;Hras*^*V12*^ iBMK (*Atg5*^−*/*−^) cells (*n* = 14 mice). The respective diet regimens were continued for the duration of the study, and tumor growth was monitored for 4 weeks. Tumors were palpated and measured with Vermeer™ calipers weekly, and tumor volume was calculated using the formula 4/3п (*r*_1_)^2^(*r*_2_). Mice were fasted for 6 h, anesthetized with CO_2_ for cardiac puncture (to obtain serum for metabolomics analysis), and then subsequently killed by cervical dislocation. Tumors were harvested, weighed, and either snap frozen in liquid nitrogen and stored at −80 °C or embedded in optimal cutting temperature compound (OCT) and stored at −20 °C.

### Blood glucose and serum energy balance-related hormone analyses

Analysis of fasting glucose was performed on blood collected at the 16-week time point using a Contour glucometer and glucose test strips (Bayer HealthCare LLC, Mishawaka, IN). Serum insulin-like growth factor (IGF)-1 levels were determined by ELISA (Quantikine IGF-1 ELISA kit; R&D Systems, Inc., Minneapolis, MN), according to manufacturer’s directions. Serum levels of insulin, leptin, and adiponectin were measured using Lincoplex® bead-based assays (Millipore Corporation, Billerica, MA) on a BioRad Bioplex® analyzer (BioRad, Hercules, CA), according to manufacturer’s directions.

### Metabolomic sample preparation and magnetic resonance spectroscopy acquisition

Frozen serum samples from a random sample of 11 mice/diet group that were injected with *Atg5*^*+/+*^ tumor cells at 16 weeks of study and terminated at 20 weeks of study were deproteinized by ultrafiltration (Nanosep 3K OMEGA, Pall Corporation, MI) at 4 °C [35, 36]. Filtered serum was placed in 3 mm magnetic resonance spectroscopy (MRS) tubes (Norell, Landisville, NJ) containing phosphate buffer (final concentration 100 mM, pH 7.0), sodium 3-(trimethylsilyl)propionate-2,2,3,3-d4 (TMSP, 0.1 mM final concentration; Cambridge Isotope Laboratories, Andover MA), 0.75 % (*w*/*v*) sodium azide and 10 % D_2_O (final concentration; Cambridge Isotope Laboratories).

One-dimensional ^1^H-MRS spectra were acquired on the filtered serum samples using a 700-MHz Bruker Avance spectrometer equipped with a Bruker Triple Resonance TCI cryoprobe and SampleXpress high-throughput robotics (Bruker BioSpin Corp., MA). Each sample was allowed to equilibrate for 5 min inside the probe before starting data acquisition. The acquisition parameters for 1D spectra were a 90° flip angle, 6-kHz spectral width, 1-s relaxation delay, 32,000 data points, 8 dummy scans, and 6-kHz spectral width. Excitation sculpting pulse sequence was implemented to suppress the water signal [37].

### Histopathologic and immunohistochemical analyses

Tumors were fixed in formalin, embedded in paraffin, cut into 4-μm thick sections, placed on glass slides (one section/slide), and processed for either hematoxylin and eosin (H&E) or immunohistochemical (IHC) staining. Slides were deparaffinized in xylene and hydrated, and antigens were retrieved by microwaving slides for 10 min with 10 mM citrate buffer. Endogenous peroxidase activity was quenched by exposing slides to 3 % hydrogen peroxide for 10 min. Following 30 min blocking with Biocare blocking reagent (Biocare Medical, Concord, CA) was followed by incubation with primary antibody diluted in the same blocking buffer as follows: Ki-67 (Dako, Carpenteria, CA; 1:200, 4 °C overnight); slides were washed with PBS, secondary antibody was applied for 30 min at RT, followed by another three washes with PBS. Diaminobenzidine (DAB) was used to develop the antibody stain followed by a hematoxylin counterstain to visualize nuclei. Slides were scanned and digitized using the Aperio Scanscope System (Scanscope XT; Aperio Technologies, Vista, CA).

### Analysis of autophagy

In order to assess autophagy activation, *Atg5*^*+/+*^ H-Ras-expressing iBMK cells were transfected with a GFP/RFP-LC3 plasmid prior to implantation into nude mice. The extent of fluorescence and number of membrane-bound RFP-LC3 puncta per field in each tumor section was quantified using a fluorescence microscope as previously described [28]. For in vitro analysis of autophagic flux, cells were incubated with 10 μM chloroquine for 24 h before being lysed using radioimmunoprecipitation buffer (RIPA), resolved by SDS-PAGE, transferred to a PVDF membrane and probed with antibodies specific for LC3B (Cell Signaling #2775), Atg5 (Cell Signaling #12994), or β-Actin (Santa Cruz #).

### Data analysis

Data are reported as mean ± standard error of the mean (SEM). Comparisons between the diet groups with respect to body composition parameters, serum markers, and tumor weight differences were performed using *t* test. Changes in tumor volume between diet groups and tumor type were assessed using repeated measures analysis. The proportion of mice with palpable tumors between the autophagy-competent and autophagy-deficient tumors was compared using Fisher’s exact test. With the exception of the MRS data, statistical analyses were conducted using either Microsoft Excel software or STATA software (College Station, TX). All *t* tests were two-tailed, and results were considered significant if *p* < 0.05.

All the MRS datasets were processed using MetaboLab [38] in the MATLAB programming environment (MathWorks, Inc., Natick, MA). MRS resonances were assigned and the metabolites quantified using the Chenomx NMR Suite and other available libraries [39–42]. Principal component analysis (PCA) of ^1^H-MRS spectra of serum from control and CR mice was performed using STATA.

## Results

### Effects of CR on body composition parameters, blood glucose, and serum energy balance-related hormones in nude mice

**Fig. 1:**
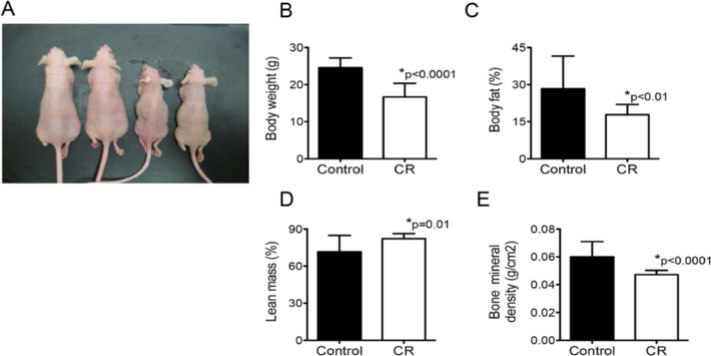
Effects of CR on body composition. Nude mice were fed a control or CR diet for 16 weeks (*n* = 19/diet group), and body size (**a**), body weight (**b**), percent body fat (**c**), percent lean mass (**d**), and bone mineral density (**e**) were compared. Data presented are the mean ± SEM

A total of 94 female athymic nude mice were administered either a control or CR diet, including a subset of mice (*n* = 19/diet group) killed at 16 weeks on diet, in order to analyze body composition and serum profiles. Administration of the diet regimens for 16 weeks induced two different body phenotypes in the nude mice (Fig. 1a). Relative to the control diet-fed mice, the CR mice weighed significantly less (*p* < 0.0001; Fig. 1b); had decreased body fat and bone mineral density (*p* < 0.01 and *p* < 0.0001, respectively; Fig. 1c, e); and had greater lean mass (*p* = 0.01; Fig. 1d).

**Fig. 2:**
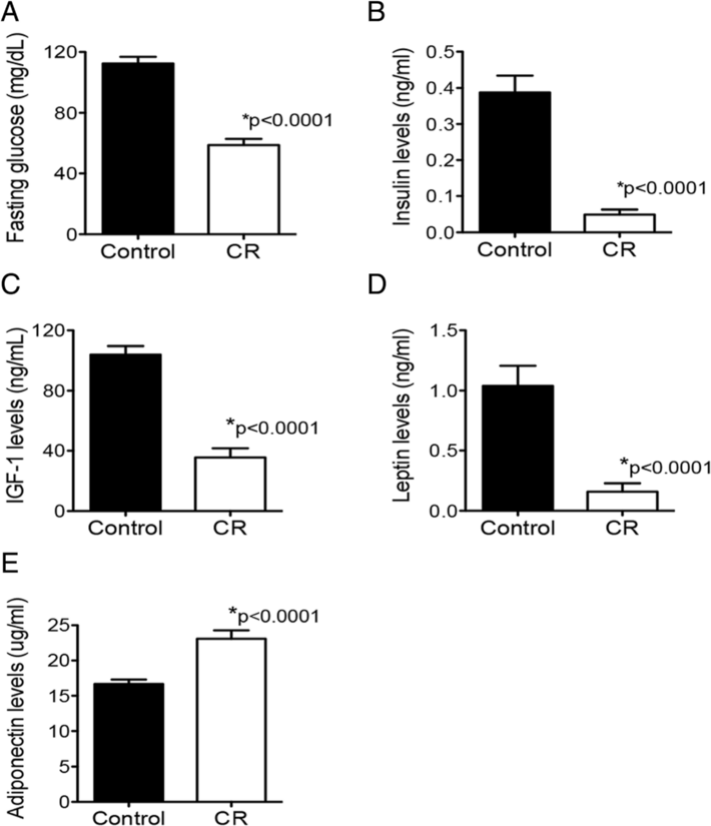
Effects of CR on blood glucose and serum energy balance-related hormones. Whole blood was collected from a subset of mice on CR or control diet for 16 weeks and analyzed for blood glucose concentration (**a**). Serum levels of insulin (**b**), IGF-1 (**c**), leptin (**d**), and adiponectin (**e**) were measured using a BioRad BioPlex analyzer. Data presented are the mean ± SEM (*n* = 11/diet group)

CR mice displayed significantly lower levels of fasting blood glucose (Fig. 2a), serum insulin (Fig. 2b), serum IGF-1 (Fig. 2c), and serum leptin (Fig. 2d) than the control group, while levels of circulating adiponectin were significantly increased by CR (Fig. 2e; *n* = 11 randomly selected samples/diet group and *p* < 0.0001 for all analytes).

### Effect of CR and autophagy inhibition on tumor growth

**Fig. 3:**
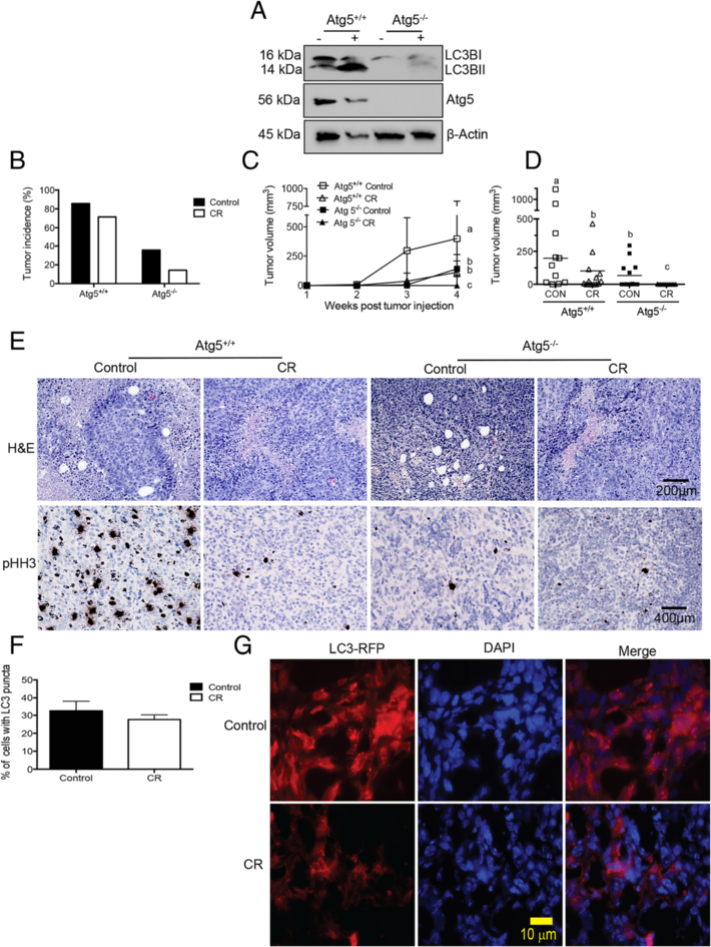
Autophagy inhibition and CR reduce tumor growth. **a** Immunoblot analysis of Atg5 expression and LC3B cleavage in *Atg5* ^*+/+*^ *;Hras* ^*V12*^ iBMK cells or *Atg5* ^−*/*−^ *;Hras* ^*V12*^ iBMK cells incubated with or without 10 μM chloroquine (CQ). *Atg5* ^*+/+*^ or effect of autophagy inhibition and CR on tumor incidence (**b**), tumor growth (**c**), and final tumor volume (**d**). *Atg5* ^−*/*−^ mice were transplanted into nude mice fed either a control or CR diet (*n* = 14/diet and genotype), and tumor volume was measured weekly. **e** Representative H&E (i) and Ki67 IHC staining (ii) images of *Atg5* ^*+/+*^ and *Atg5* ^−*/*−^ tumors from mice fed a control or CR diet. **f** Effect of CR, relative to control diet, on LC3 puncta formation and RFP-LC3 fluorescence in tumors from *Atg5* ^*+/+*^ *;Hras* ^*V12*^ iBMK cell transplants. **g** Fluorescence microscopy of RFP-LC3 expressing *Atg5* ^*+/+*^ tumor slices from mice fed a control or CR regimen. Graphs presented are mean ± SEM and values with different letters are significantly different at *p* <0.05

Following 16 weeks of diet regimens, all remaining mice (*n* = 56) were injected subcutaneously with either autophagy-proficient *Atg5*^*+/+*^*;Hras*^*V12*^ iBMK cells or autophagy-deficient *Atg5*^−*/*−^*;Hras*^*V12*^ iBMK cells (14 mice/cell line for each diet). These iBMK cells were derived from *Atg5*^*+/+*^ and *Atg5*^−*/*−^ mice, respectively. Previous studies established that deletion of *Atg5* results in a profound autophagy defect [28, 43]. Atg5 deletion and autophagy deficiency was confirmed by western immunoblot analysis for Atg5 and LC3B (Fig. 3a). Unlike *Atg5*^*+/+*^ cells, incubation with the lysosomal inhibitor chloroquine does not cause accumulation of LC3B-II in *Atg5*^−*/*−^ cells (Fig. 3a).

As shown in Fig 3b, tumor incidence 4 weeks after injection was significantly (*p* < 0.05) higher in control diet-fed mice with *Atg5*^*+/+*^ tumors (12/14, 86 %) than *Atg5*^−*/*−^ tumors (5/14; 36 %). In CR-fed mice, *Atg5*^*+/+*^ tumor incidence was 71 % (10/14;) and *Atg5*^−*/*−^ tumor incidence was 14 % (2/14). Tumor growth (Fig. 3c) and final tumor volume (Fig. 3d) was greatest for *Atg5*^*+/+*^tumors in control-fed mice, intermediate for *Atg5*^*+/+*^tumors in CR-fed mice and *Atg5*^−*/*−^ tumors in control-fed mice, and lowest for *Atg5*^−*/*−^ tumors in CR mice.

Tumors excised from CR groups displayed reduced intratumoral adipocytes compared to those from control groups (Fig 3e [i]), and both CR and autophagy deficiency was sufficient to reduce proliferative (Ki67 positive) cells within the tumor (Fig 3e [ii]).

### Effect of CR on autophagic flux in tumors

No diet-dependent difference in the percent of cells with LC3 puncta was detected in tumors obtained 4 weeks after mice were transplanted with *Atg5*^*+/+*^ tumor cells (Fig. 3e). However, RFP-LC3 fluorescence was significantly decreased in tumors from CR mice compared with control-fed mice (Fig. 3f). Autophagy is a highly dynamic process, and the flux of RFP-LC3 puncta formation and autophagosome degradation at low pH is rapid [44]. Increased RFP-LC3 turnover due to increased autophagic flux in response to CR likely explains the observed reduction in RFP-LC3 fluorescence in the *Atg5*^*+/+*^ tumors from CR mice.

### Effect of CR on serum metabolomic profile

**Fig. 4:**
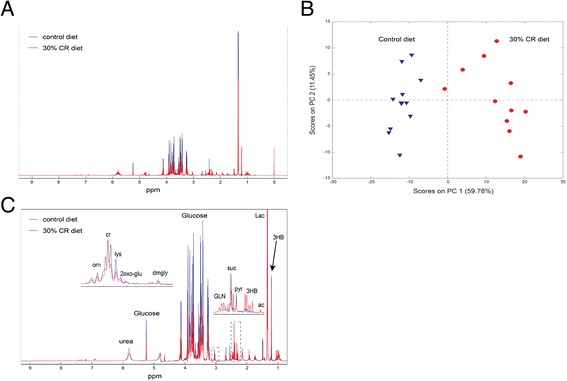
Effect of CR on serum metabolomic profile. **a** Representative 1D ^1^H-MRS spectra of serum isolated from mice fed a control (*n* = 11) or CR (*n* = 11) diet for 20 weeks and transplanted with *Atg5* ^*+/+*^ cells. **b** Scores plots from principal component analysis (PCA) of ^1^H-MRS spectra from control and CR serum. **c** Differential metabolites identified in PCA between diet groups are indicated on ^1^H-MRS spectra

**Fig. 5:**
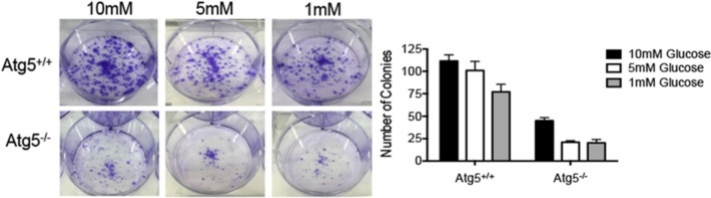
Effect of glucose restriction on colony formation and growth of *Atg5* ^*+/+*^ and *Atg5* ^−*/*−^ cells. **a** Representative images of colonies formed by *Atg5* ^*+/+*^ and *Atg5* ^−*/*−^ cells grown in media containing 10, 5, or 1 mM glucose. Graph is mean ± SEM and is inclusive of three independent experiments

The low rate of tumor development and growth precluded further in vivo analysis of the effect of CR in *Atg5*^−*/*−^ tumors, although previous studies have established that LC3 puncta formation is negligible in *Atg5*^−*/*−^ tumors [28]. To establish a relevant in vitro approach to study the effects of CR and/or autophagy inhibition in *Atg5*^−*/*−^ and *Atg5*^+/+^ cells, we first characterized the impact of CR on the serum metabolomic profile using an untargeted MRS-based metabolomics approach was evaluated in serum samples obtained 4 weeks after mice were transplanted with *Atg5*^*+/+*^ tumor cells. Figure 4a presents representative magnetic resonance spectra. PCA was performed on the entire MRS dataset (*n* = 11 serum samples per group) to evaluate the global metabolic changes induced by CR relative to control (Fig. 4b); the scores plot indicates that the serum samples from control and CR mice are characterized by distinct metabolic profiles. A further analysis of the PCA loadings plot in conjunction with the MRS spectra profiles led to the identification of several discriminant metabolites that significantly change in the serum profile of CR mice. The average serum concentration change (as percent change in CR mice compared to control) of the identified, statistically significant (*p* value after positive false discovery rate correction <0.05) metabolites are shown in Table 1 and highlighted in the MR spectra in Fig. 5c. The serum concentrations of glucose, several amino acids, and Krebs cycle intermediates decreased as a consequence of CR, while ketone bodies (acetoacetate and 3-hydroxybutyrate in particular, and also acetone) increased, suggesting CR resulted in a metabolic switch away from glucose metabolism.

**Table 1:** Effect of CR on serum metabolites

Metabolite	% of control serum	*P* value after pFDR correction
Glucose	42.0	5.5E−08
Lysine	47.2	2.4E−07
Lactate	50.8	3.1E−07
Alanine	52.0	5.53E−08
Pyruvate	57.3	5.6E−06
Tyrosine	57.7	1.07E−06
Fumarate	60.4	0.008
2-Oxoglutarate	61.9	0.023
Ornithine	66.1	0.018
Acetoacetate	842.2	4.1E−04
3-Hydroxybutyrate	500.2	4.1E−04
Urea	164.8	0.004
Acetone	152.8	0.017

Values represent the average serum concentration change (as percent change in serum from CR mice relative to control-fed mice) of the identified, statistically significant metabolites (Fig. 4c), based on *p* values after positive false discovery rate (pFDR) correction <0.05

### Effect of glucose deprivation on colony formation

*Atg5*^*+/+*^ and *Atg5*^−*/*−^ cells were grown in media with normal or reduced glucose concentrations to model in vitro the metabolic state induced by control and CR diets in vivo (Fig. 5). *Atg5*^−*/*−^ cells formed fewer colonies than *Atg5*^*+/+*^ in all conditions, and autophagy deficiency (*Atg5*^−*/*−^) combined with low glucose (5, 1 mM) resulted in significantly reduced colony formation.

## Discussion

Our findings confirm the hypothesis that combining the systemic metabolic reprogramming that occurs with CR with cell intrinsic autophagy inhibition exerts more potent anticancer effects in vivo and in vitro than either CR or autophagy inhibition alone. We found that administration of a 30 % CR diet to nude mice (in which CR has not been previously well studied) resulted in a significant decrease in body fat, fasting blood glucose, and serum insulin, IGF-1, and leptin levels, while it increased adiponectin levels. Metabolomic analyses of CR-fed nude mice showed a significant decrease in circulating glucose, several amino acids (including alanine, pyruvate, ornithine, and tyrosine), and several Krebs cycle intermediates (including fumarate, 2-oxoglutamic acid) compared to control mice. There was also a concomitant increase in metabolites indicating CR induced negative energy balance, increased fatty acid oxidation, and ketosis, including acetoacetate, 3-hydroxybutyrate, urea, and acetone, consistent with a metabolic shift away from glucose metabolism and toward fat/ketone metabolism during CR.

To assess the separate and interactive effects of CR and autophagy deficiency on tumor development and growth, we used a xenograft model in nude mice subcutaneously injected with iBMK cells derived from *Atg5*^*+/+*^ or *Atg5*^−*/*−^ mice, immortalized by E1A and dominant-negative p53 expression, stably transfected with a H-Ras^G12V^ expression construct, and a p-tFl-RFP-tandem tagged LC3 expression plasmid [28]. CR, relative to control diet, significantly reduced the growth of transplanted *Atg5*^*+/+*^ tumors. Similar to CR, autophagy deficiency (*Atg5*^−*/*−^) decreased tumor growth in control-fed mice. Tumor growth was lowest in the combined CR and autophagy-deficient group. Tumors derived from mice transplanted with autophagy-competent (*Atg5*^*+/+*^) cells and fed a CR diet displayed increased autophagic flux. Despite several limitations, including the relatively small sample size (*n* = 14 mice/diet group for each cell line), the low incidence of *Atg5*^−*/*−^ tumors, and the shortcomings associated with xenograft models for studies of diet and cancer [20], these data illustrate three important characteristics of energy/nutrient dependence of many tumors that could inform new strategies for cancer prevention and treatment. First, intracellular sources of nutrients (autophagy) are required for efficient tumor growth. In addition, restriction of external substrate (via CR) significantly prohibits tumor growth. Finally, restricting the ability of cells to harness intracellular substrates under conditions of limited external energy and nutrients may have potent anticancer effects.

Metabolic reprogramming is a hallmark of tumorigenesis, and reduced reliance on mitochondrial respiration and increased glycolysis are common in many cancers [45, 46]. Associated with this so-called Warburg effect is increased dependence on adequate levels of systemic glucose and other substrates. In this study using metabolomics profiling, we find that mice fed a CR regimen had reduced serum levels of glucose and several amino acids. Reduced circulating glucose and amino acids decreases the external availability of these nutrients to the developing cancer cells, effectively starving the tumor via the external environment. The striking increase of ketone bodies indicates increased fatty acid breakdown as an alternative to carbohydrate metabolism as a primary source of energy in response to CR. As low carbohydrate/high fat diets, which rewire energy metabolism to utilize ketones, are effective in reducing tumor growth in a number of cancer models [47–49], our findings suggest that the beneficial anticancer effects of both CR and ketogenic diets may have common underlying mechanisms.

The observed suppressive effects of CR on tumor growth in this model are consistent with several previous studies in other cancer models [3–6, 15]. CR is known to induce autophagy in a number of normal tissues and organs [50, 51], and autophagy activation can promote survival in cancer cells during nutrient deprivation [52–55]. Our observed inhibitory effect of genetically induced autophagy deficiency on tumor development and growth is also consistent with previous reports of the anticancer effects of autophagy inhibition [23, 25–31]. However, to date, little research has explored the potential of combining dietary (such as CR) or other interventions that induce negative energy balance with autophagy inhibition as a therapy in the treatment of solid tumors.

One report by Harhaji et al. showed that combining chloroquine (which inhibits autophagy by interfering with lysosome function) with serum starvation resulted in greater growth arrest and cell death relative to either treatment alone in B16 melanoma cells, U251 glioma cells, and L929 fibrosarcoma cells in vitro [56]. Moreover, consistent with our in vivo finding, Harhaji et al. showed that autophagy inhibition (again via chloroquine) and a 30 % total diet restriction in vivo each slowed the growth of transplanted B16 melanoma cells in C57BL/6 mice, and combining chloroquine treatment with the diet restriction inhibited the development and growth of B16 melanoma better than either intervention alone [56]. The in vivo study of B16 melanomas was small (eight mice/treatment group), involved a 30 % total diet restriction (which unlike a CR regimen is subject to vitamin and mineral deficiencies) and did not include analysis of autophagy in the tumors. Nonetheless, the Harhaji et al. study supports our finding that combining autophagy inhibition with CR may be an effective anticancer strategy.

Although to our knowledge there are no other reports in the literature combining CR and autophagy inhibition, further support for this approach of combining autophagy induction in cancer cells with systemic metabolic alterations comes from a study assessing the growth of A-2058 human melanoma cells grown in nude mice receiving a control or leucine-restricted diet, with or without chloroquine treatment [57]. Chloroquine treatment alone had a modest effect on tumor growth, while leucine deprivation alone had little effect on tumor growth. However, the combination of leucine deprivation and chloroquine treatment strongly suppressed melanoma growth [57].

## Conclusions

In a murine model of Ras-driven cancer, combined restriction of extracellular (via CR) and intracellular (via autophagy inhibition) sources of energy and nutrients more potently suppresses tumor growth than either CR or autophagy inhibition alone. Similarly, glucose deprivation combined with autophagy deficiency reduced in vitro colony formation more potently than either glucose or autophagy deficiency alone. Thus, interventions targeting both systemic energy balance and tumor-cell intrinsic autophagy may represent a potent anticancer strategy. Current efforts in our laboratory using multiple preclinical models of breast, colon, and pancreatic cancer focus on elucidating the mechanisms underlying the separate and combined effects of CR (or other forms of nutrient deprivation) and autophagy inhibitors, including several emerging autophagy inhibitors currently under clinical development [58].

## Abbreviations

CR: Calorie restriction
ATG5: Autophagy-related protein 5
IGF-1: Insulin-like growth factor 1
iBMK: Immortalized baby mouse kidney epithelial cells
LC3: Microtubule-associated protein1 light chain 3
AMPK/mTOR: AMP-activated protein kinase/mammalian target of rapamycin
OCT: Optimal cutting temperature compound
RIPA: Radioimmunoprecipitation
DAB: Diaminobenzidine
MRS: Magnetic resonance spectroscopy
PCA: Principal component analysis
pFDR: Positive false discovery rate
